# Female obesity: clinical and psychological assessment toward the best treatment

**DOI:** 10.3389/fendo.2024.1349794

**Published:** 2024-05-03

**Authors:** Valeria Guglielmi, Riccardo Dalle Grave, Frida Leonetti, Anna Solini

**Affiliations:** ^1^ Unit of Internal Medicine and Obesity Center, Department of Systems Medicine, Policlinico Tor Vergata, University of Rome Tor Vergata, Rome, Italy; ^2^ Department of Eating and Weight Disorders, Villa Garda Hospital, Garda, VR, Italy; ^3^ Department of Medical-Surgical Sciences and Biotechnologies, Sapienza University of Rome, Rome, Italy; ^4^ Department of Surgical, Medical, Molecular and Critical Area Pathology, University of Pisa, Pisa, Italy

**Keywords:** obesity, gender, females, psychological characteristics, complications, behavioural and pharmacological treatment

## Abstract

Obesity is a heterogeneous condition which results from complex interactions among sex/gender, sociocultural, environmental, and biological factors. Obesity is more prevalent in women in most developed countries, and several clinical and psychological obesity complications show sex-specific patterns. Females differ regarding fat distribution, with males tending to store more visceral fat, which is highly correlated to increased cardiovascular risk. Although women are more likely to be diagnosed with obesity and appear more motivated to lose weight, as confirmed by their greater representation in clinical trials, males show better outcomes in terms of body weight and intra-abdominal fat loss and improvements in the metabolic risk profile. However, only a few relatively recent studies have investigated gender differences in obesity, and sex/gender is rarely considered in the assessment and management of the disease. This review summarizes the evidence of gender differences in obesity prevalence, contributing factors, clinical complications, and psychological challenges. In addition, we explored gender differences in response to obesity treatments in the specific context of new anti-obesity drugs.

## 1 Introduction

Obesity is a chronic progressive and relapsing disease (1) associated with reduced quality of life, increased morbidity, disability, and lower life expectancy (2). Although obesity has reached epidemic proportions worldwide, impacting 650 million adults globally (3), only in recent years, obesity heterogeneity has been recognized, and sex/gender differences increasingly recognized in several aspects of the disease, including overall prevalence and complications.

However, sex/gender is still rarely considered in the assessment and management of the disease.

Thus, this review aimed to summarize the evidence of gender differences in obesity prevalence, contributing factors, clinical complications, and psychological challenges, but also to explore gender differences in response to obesity treatments in the specific context of new anti-obesity drugs.

## 2 Obesity: nods of epidemiology and etiopathogenesis

In contrast to overweight, obesity is more prevalent among females (24%) compared to males (22%), with even greater differences in certain regions. Although obesity prevalence is globally higher in people with lower educational attainment, gender analysis shows heterogeneity based on socioeconomic status as defined by income, education, employment status, and place of residence (4). For example, in low- and middle-income countries, obesity is prevalent in women of higher socioeconomic classes, but in economically developed countries, obesity mainly affects women of lower socioeconomic status (5), providing epidemiologic evidence of the ‘obesity transition’ model (6).

The differences, as mentioned above, in obesity prevalence between men and women may be in part explained by gender norms and identity that condition eating habits and physical activity behaviors. Indeed, gender-based differences in eating behaviors are already reported by school age (7), and greater preference for foods high in sugar (8) and a stronger association of stress-related eating with weight gain have been described in women (9, 10). Other factors may be represented by sex hormones and physiological events during the women’s life course, like pregnancy and menopause (11, 12). Additionally, neuroimaging studies found disparities between genders in structural and functional obesity-related brain alterations (13) with stronger neural response to food-related stimuli in women (14, 15).

In women’s life course, maternal obesity has gained growing attention not only for increasing the risk of pregnancy complications like gestational diabetes, hypertension, and preeclampsia but also for affecting fetal growth and for being an independent risk factor for childhood and adult obesity (16).

Although changes in the environment have undoubtedly led to the steep increase in obesity prevalence by influencing poor quality food choices, overeating, unlimited availability of food, and sedentary lifestyle (17), the etiopathogenesis of obesity is complex, resulting from an interaction between environmental and innate biological factors.

Twin, family and adoption studies have highlighted a strong heritability component to BMI, which varies between 40% and 70% (18). After that, more than 100 susceptibility genes associated with obesity have been identified in genome-wide association studies (GWAS) (19). Individuals who inherit larger subsets of susceptibility genes are more prone to gain weight in response to the ‘obesogenic’ environment (20). Namely, gene-environment interactions generate a unique human biological and behavioral interface that thoroughly explains the interindividual variations of body weight response to an identical environment.

Thus, obesity extends far beyond individual behavior (21) and the energy balance equation of ‘calories in’ and ‘calories out’ progressively emerged as an oversimplification of its complex pathogenesis.

Currently, the neuronal pathways that control the homeostatic and hedonic aspects of food intake have been recognized as major players in the development of obesity. Notably, in one of the largest GWAS in terms of both individuals and single nucleotide polymorphisms (SNPs) studied, the 97 loci associated with BMI that were identified were all expressed in the brain (22), consistent with the observation that even the monogenic forms of obesity so far described are caused by genetic defects affecting the central regulation of appetite and satiety (23), and therefore supporting the primacy of the central nervous system in the maintenance of energy homeostasis. The homeostatic pathway relies on proopiomelanocortin pro-opiomelanocortin (POMC)/cocaine- and amphetamine-regulated transcript (CART) and neuropeptide Y (NPY)/agouti-related peptide (AgRP) neurons of the arcuate nucleus of the hypothalamus and controls energy balance by increasing hunger after weight reduction. It is an archaic system that evolved over ages and is characterized by scarce food availability. Consequently, it results in an imbalance toward energy conservation and is maladaptive to the current ‘obesogenic environment’. Instead, the hedonic control system of food intake, coordinated by the nucleus accumbens and the mesolimbic dopaminergic system, focuses on the reward associated with food consumption regardless of energy needs (24).

In patients with obesity, as a major consequence of gene-environment interactions, the homeostatic system is dysregulated and results in an increased food intake that sustains and maintains excess adiposity (25).

The pathophysiology of obesity is further operational in response to a weight loss, explaining why body weight tends to be regained over time. Indeed, a high equilibrium level of body fat mass (also termed “set point”) is an unrelenting feature of obesity and an integral part of its pathophysiology (19, 26). Consequently, weight loss would trigger hormonal and neurotransmitter homeostatic responses, which determine an increase in appetite and reduced energy expenditure (27) until weight is regained (28). Even the hedonic system drives the weight back by increasing the desire for foods with greater caloric density and high fat and sugar content.

Sexual asymmetry is evident in the organization of the POMC system. Indeed, female brain presents increased POMC neuronal fibers, higher levels of the POMC protein and decreased NPY expression compared to males. Accordingly, while testosterone stimulates NPY expression in the hypothalamus in males, in females estradiol impairs the excitability of the NPY neurons (29). In addition, leptin concentrations are four times higher in women (30), and in rat models female brain is more sensitive to leptin, while male brain is more sensitive to insulin (31).

Neuroimaging studies also helped identify gender differences in obesity-associated structural and functional alterations throughout the brain. Indeed, male obesity appears to be associated to more evident changes in cortical somatosensory regions, whereas reward regions appear to be more involved in female obesity, consistent with distinct neural activation pattern in response to food-related stimuli for each sex/gender (13, 14).

To add complexity to understanding obesity pathogenesis, nutritional and environmental factors can influence biology through epigenetic changes. These alterations in the DNA and histone structure, which can be inherited or arise *de novo* in a tissue-specific manner, alter gene expression persisting across generations (32, 33). In this view, growing evidence has indicated that excess maternal body weight or weight gain during pregnancy participates in the programming of body weight and metabolism in offspring by epigenetics, for long affecting their homeostatic control system of energy balance (34).

The ‘obesogenic’ environment can also alter metabolic function and homoeostasis by modifying gut microbiota diversity and composition (35). Obesity-associated microbiota influences the efficiency of calorie uptake from ingested foods, host energy harvesting, insulin resistance, hepatic metabolism, inflammation, as well as central regulation of appetite and satiety and food reward signalling, which all have crucial roles in obesity. Moreover, some strains of bacteria and their metabolic products might directly target the brain by vagal afferent innervation or immune-neuroendocrine mechanisms (36, 37).

## 3 Assessment of obesity by gender

The value of the BMI for tracking trends of weight in the population and identifying health risks is widely recognized (38). However, the BMI has serious limitations for individuals due to its high specificity but low sensitivity in identifying excess adiposity (39), accounting for only 25% of body fat variance in both sexes (40). Consequently, although clinical guidelines suggest using BMI as a starting point, whenever it is above the appropriate ethnic cut-offs (41, 42) body fat should be assessed by bioelectric impedance and Dual X-ray absorptiometry (DXA). The use of magnetic resonance imaging (MRI), computed tomography (CT), or more advanced scanning methods (43), although providing the most accurate measurements, is limited in clinical practice by costs and viability.

Another limitation of BMI is that it does not provide any information on abdominal fat distribution, which is crucial in determining obesity-related health risks. Waist circumference (WC) is the measure of choice to diagnose central obesity as compared to other combined indices, including waist-to-hip ratio (WHR), weight-to-height ratio (Wt/Height), or waist-to-height^0.5^ ratio (WC/Height^0.5^), whose predictive value highly differs for the various markers of cardiometabolic risk (44). However, it should be noted that WC, though improving the predictive value of BMI, remains a suboptimal predictor of mortality (45, 46).

Anthropometric measures and body composition analysis allow us to appreciate gender differences, with women having greater fat mass and a relatively more peripheral distribution of adiposity. These differences are already evident in early life but become much more apparent in adolescence due to an increase in steroid hormone concentrations. Accordingly, parity and menopause have been related with changes in body composition (47) and, in particular, with gains in visceral and central adiposity (48) (49). However, differences in WC between adult men and women are seen at all ages and levels of fatness, fully justifying the current practice of using different waist thresholds for men and women.

Age-related changes in body composition may increase fat mass and decrease muscle mass (50) in both males and females, with men typically experiencing a faster decline in muscle mass (51). Accordingly, in the community-dwelling elderly population, men are more likely than women to have sarcopenia (52), a condition characterized by age-related low muscle strength, quantity and/or quality, and reduced functional performance (53) driven mainly by hormonal changes, nutritional factors, inflammation and disease states. Despite its lower prevalence, higher mortality risk is conferred by sarcopenia in women (54). Sarcopenic obesity, defined by the coexistence of both sarcopenia and obesity, further increases morbidity, disability, and mortality than obesity or sarcopenia alone, so the assessment of indices of sarcopenia is highly recommended (55).

Nevertheless, as mentioned above, neither BMI nor other anthropometric measurements fully capture the heterogeneity of obesity, which is instead more comprehensively assessed using the Edmonton Obesity Staging System (EOSS), a validated 5-stage system based on obesity-related medical, physical, and psychological impairments. Increasing EOSS severity strongly correlates with mortality (45, 56) and adverse outcomes like postoperative complications (57), and is associated with increased health service and multiple-drug use and less weight loss (56, 58). In women with obesity, EOSS may also be helpful to estimate the risk of cesarean delivery after labour induction (59) and pregnancy rates after fertility treatments (60). Thus, EOSS may influence patients’ management and guide treatment prioritization (61, 62).

## 4 Clinical complications

Obesity is associated with an increased risk of all-cause mortality, with cardiovascular disease (CVD) and malignancy as the most common causes of death (63).

The excess adiposity can cause clinical complications through anatomical, load-bearing and metabolic effects. Also, fat distribution, genetics, lifestyle and psychological factors play a role in the propensity to develop different obesity complications.

**Figure 1:**
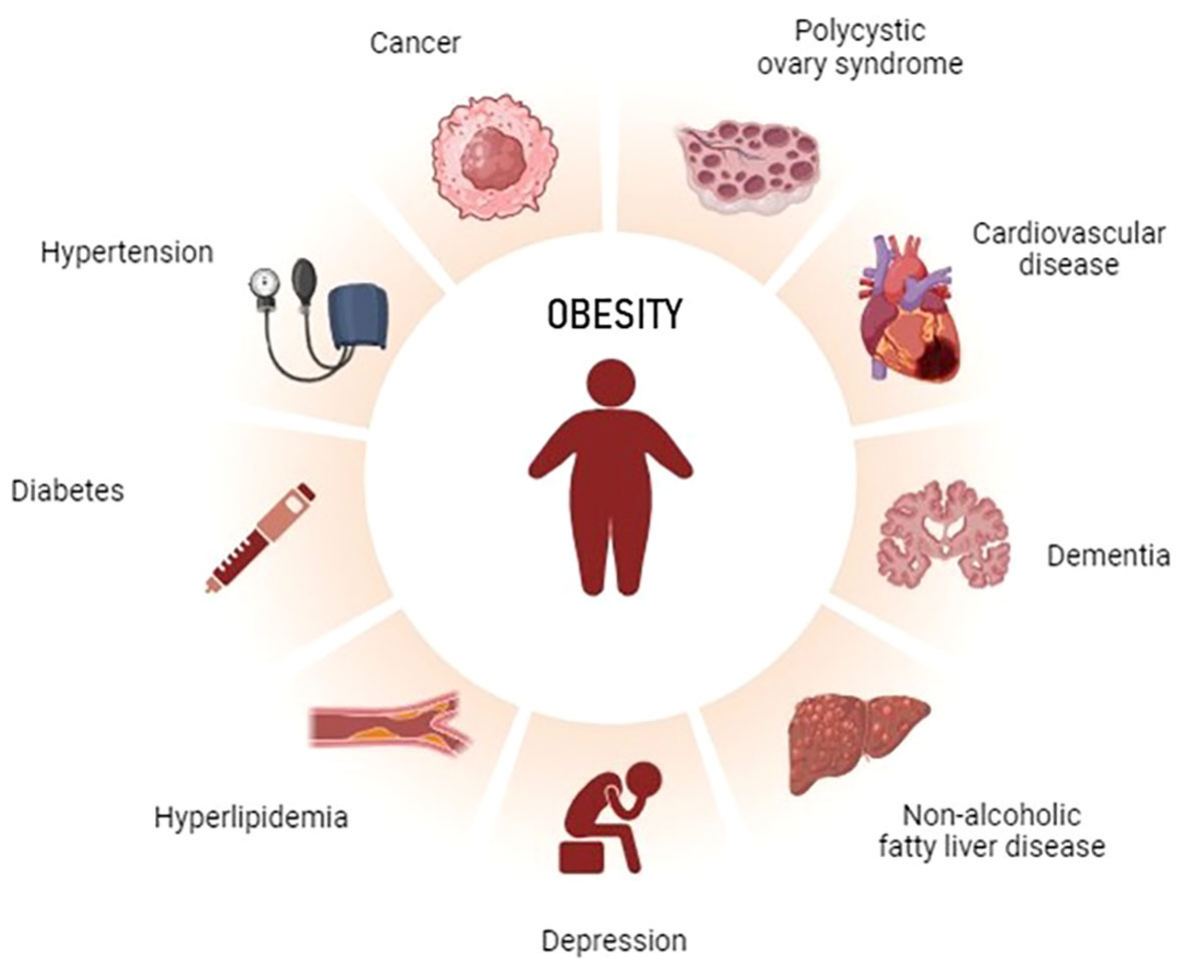
Clinical complications of obesity that may differently affect males and females. Created with BioRender.com.

In fact, obesity can present with various disease burden and lead to many clinical complications that require an individualized assessment (**Figure 1**).

### 4.1 Type 2 diabetes

Obesity and type 2 diabetes (T2D) are strongly linked. Indeed, more than 80% of people with T2D have a BMI>25 Kg/m^2^, and more than 90% have a waist circumference above the normal limits (64). The prevalence of T2D in the general population is around 6%, while in the population affected by obesity it rises up to 20% (65). The risk of developing T2D is 6- and 12-fold increased in men and women with obesity, respectively. In recent years, this strong association between obesity and T2D has led to define a new condition called “diabesity”, so that testing for T2D should be considered at any age in adults with overweight or obesity who have one or more of the risk factors (**Table 1**) (66).

**Table 1:** Risk factors for type 2 diabetes.

First-degree relative with diabetes
High-risk race/ethnicity (African American, Asian American, Latino, Native American; Pacific Islander)
Hypertension (> 140/90 mmHg or on therapy for hypertension)
HDL cholesterol level < 35 mg/dl and/or triglycerides level > 250 mg/dl
Women with polycystic ovary syndrome
Physical inactivity
The presence of other conditions of insulin resistance

From ref. (66).

In randomised controlled trials, lifestyle interventions for weight loss resulted in several metabolic benefits, such as decreased insulin resistance, blood pressure, inflammation, incidence of T2D and improved lipid profiles and glycaemic control in subjects with diabetes. Over 15% weight loss can lead to remission of T2D, especially when diabetes duration is short, heart failure improvements, and cardiovascular mortality reductions (67, 68).

The results of the *post-hoc* analysis of the Look AHEAD Study, involving also a population of 5,145 adults with diabetes randomly assigned to an intensive intervention or lifestyle control, suggest a strong association between the extent of weight loss and incidence of CVD in people with T2D. Furthermore, a close correlation between weight loss and the incidence of diabetes have been shown, underlining the need for intervention on excess weight, particularly in people with diabetes, dyslipidemia, and hypertension (69).

Besides lifestyle modifications and pharmacotherapy, for individuals with a BMI > 40 Kg/m^2^ or BMI > 35 Kg/m^2^ with complications bariatric surgery (BS) represents another therapeutic option. Standard bariatric operations, including bilio-pancreatic diversion, sleeve gastrectomy (SG), Roux-en-Y gastric bypass (RYGB), and adjustable gastric banding, benefit individuals' metabolic profiles to varying degrees (70). It is well known that the benefits of BS go beyond weight loss. Namely, BS reduces chronic inflammation, alters gut hormones and microbiota promoting long-term remission of T2D (71). Indeed, in patients with a duration of T2D not exceeding 8-10 years, the possibility to get a complete remission is very high with both restrictive interventions, such as SG (72), and malabsorption ones (73). These results have contributed to hypothesize that a chronic disease like T2D could be considered reversible.

### 4.2 Gastrointestinal disorders

Central obesity is closely linked with Non-Alcoholic Fatty Liver Disease (NAFLD) development which consists in a range of progressive stages of liver disease. Indeed, ectopic fat accumulation in the liver, together with hepatic inflammatory changes and insulin resistance, drives the progression from simple steatosis to non-alcoholic steatohepatitis (NASH) which can ultimately lead to liver cirrhosis, liver failure and hepatocellular carcinoma.

Diagnostic practice includes clinical parameters, serum-based tests or imaging (such as ultrasound scan, vibration-controlled transient elastography or magnetic resonance elastography), although histologic evaluation with liver biopsy remains the gold standard to diagnose NAFLD (74). Multiple noninvasive tests, which are based on clinical and biochemical parameters including waist circumference, BMI, platelet count, albumin, liver enzymes and other criteria for metabolic syndrome, have been proposed to screen for NAFLD and NASH but have modest accuracy. In recent years, an international panel of experts has proposed a new definition for NAFLD: metabolic dysfunction-associated fatty liver disease (MAFLD) (75). The diagnosis of MAFLD is based on the presence of hepatic steatosis (detected by serum biomarker scores, imaging techniques, or liver biopsy) along with any of the following three metabolic conditions: overweight/obesity, T2D, or evidence of metabolic dysregulation (defined by at least two factors among increased waist circumference, hypertension, hypertriglyceridemia, low serum HDL-cholesterol levels and impaired fasting glucose). Hence, based on this new definition, MAFLD can be diagnosed regardless of daily alcohol consumption and other liver diseases, and further studies will need to investigate the impact of MAFLD on the progression versus cirrhosis.

The prevalence of NAFLD is higher in men up to middle age because pre-menopausal women appear to be relatively protected. However, this protective capacity is lost in post-menopause when the prevalence of NAFLD is similar in both sexes and the risk of NAFLD progression is greater in women. The sexual dimorphism in NAFLD has been increasingly attributed to beneficial actions of estrogens on lipid metabolism (76). Indeed, estrogens promote gluteo-femoral fat distribution preventing intrahepatic lipid storage and exhert direct anti-inflammatory, anti-oxidant, anti-fibrotic, anti-apoptotic properties on liver parenchyma (77). Estrogens can also beneficially modulate gut microbiota and bile acids composition (78).

Epidemiologic data have demonstrated that obesity is an important risk factor for the development of gastroesophageal reflux disease. The prevalence is proportional to the severity of obesity: 23% in individuals with a BMI<25, 27% with a BMI 25-30, and 50% with a BMI>30 (79). There is also growing evidence that obesity is associated with complications related to longstanding gastroesophageal reflux, with or without hiatal hernia, such as erosive esophagitis, Barrett’s esophagus, and esophageal adenocarcinoma mainly in the presence of central obesity (80).

### 4.3 Respiratory disease

Obstructive sleep apnea (OSA) is a sleep-related breathing disorder characterized by episodes of partial or complete collapse of the upper airway during sleep. It is common in individuals with obesity and often undiagnosed. The repetitive upper airway obstruction often results in oxygen desaturations and arousal from sleep. Symptoms of OSA may include snoring, low sleep quality with frequent arousal, daytime sleepiness, mouth dryness on awakening and headaches. OSA is an independent risk factor for several clinical consequences, including blood hypertension, CVD, stroke, and abnormal glucose metabolism potentially due to decreases in oxygenation causing oxidative stress and endothelial dysfunction. The STOP-BANG questionnaire (81) is a helpful screening tool for OSA. Although polysomnography is the “gold standard” for diagnosing OSA, it requires an overnight observation in the patient’s home, and most cases remain undiagnosed. Per each unit increase in BMI the odds of developing OSA increases by 1.14 (82).

OSA is more prevalent in men than women, but the difference reduces in older ages due to the increased prevalence of OSA in post-menopausal women (83). In fact, estrogen and progesterone are protective against OSA due to their effect on the upper-airway dilator muscles (84). Underdiagnosis of OSA in women have been also reported possibly due atypical presentation of symptoms or underreported snoring or witnessed apneas. There are also sex-related differences in polysomnographic findings such lower Apnea Hypopnea Indexes (AHIs), shorter apneic episodes, less severe desaturations in women (85).

OSA can negatively impact people’s well-being, quality of life, and work performance. It also raises the risk of road accidents by increasing the risk of falling asleep while performing daily activities. In younger women (<65 years old), OSA has also been associated with mild cognitive impairments in memory, vigilance, attention, and executive domains by causing daytime sleepiness and fostering gradual remodeling of cerebral vasculature as well as neural damage (86).

The most common treatments for symptomatic OSA are nocturnal continuous positive airway pressure (nCPAP) and weight loss (87). Pathological obesity is always associated with severe OSA, and BS is an effective method of treatment with long-term benefits. A meta-analysis comparing the effects of laparoscopic SG and RYGB on OSA demonstrated that both surgical procedures effectively reduce OSA (88).

### 4.4 Infertility

Overweight and obesity can affect reproductive health and can result in infertility in both male and female. People with excess weight, even slightly, may present difficulties in natural conception as well as in the various assisted fertilization techniques for both sexes. The most common causes of infertility are ovulatory dysfunction, male factor infertility, and tubal disease, and the principal cause of anovulation (70%) is polycystic ovary syndrome (PCOS), which occurs more frequently in women with obesity. Furthermore, the presence of BMI>27 itself is associated with anovulation independent of PCOS (89). The pathogenic mechanisms that link excess body weight to female infertility are complex and primarily due to functional alteration of the Hypothalamic-Pituitary-Ovarian Axis (HPO) axis. Obesity is frequently associated with higher circulating insulin levels, which stimulates ovarian androgen production, as well as peripheral aromatization of androgens to estrogens. Increased circulating estrogens exert negative feedback on the HPO axis, affecting gonadotropin production with consequent ovulatory dysfunction and menstrual abnormalities.

Several studies have shown that obesity negatively impacts on spontaneous pregnancy rates and increases the need to resort to assisted reproduction techniques (ART). In addition, obesity has been associated with negative fertility treatment outcomes such as the need for higher doses of gonadotrophins, increased cycle cancellation rates and fewer oocytes retrieved (90).

On the other hand, obesity may also impair male reproduction, male obesity being likewise involved in embryo quality (91) and lower success rates of ART (92) as female obesity.Obesity may negatively affects both conventional and biofunctional sperm parameters in males with obesity. A meta-analysis including 115 population-based studies reported that male partners with obesity have significantly higher infertility with an odds ratio of 1.66 compared to normal weight male partners. Most men with obesity present altered steroid synthesis and metabolism. Indeed, the visceral fat excess associated with hyperinsulinemia decrease total and free testosterone and inhibin B concentrations by promoting the conversion of testosterone into 17ß-estradiol through higher aromatase activity (93). A “metabolic” form of male hypogonadism mediated by hyperinsulinemia, inflammation, epigenetic modifications and increased nuclear and mitochondrial DNA damage, with a downstream inhibitory effect on spermatogenesis, has been described; male obesity may contribute to its development (94).

The dysregulation of sex hormones is not the only cause of infertility in males with obesity. A pivotal role is also played by heat-induced damage. Testicular thermal stress increases in men with obesity, mainly due to fat accumulation in the suprapubic region and around the pampiniform plexus. A vicious cycle is therefore present between visceral adipose tissue excess, systemic insulin resistance, and testicular malfunctioning (95).

### 4.5 Cancer

Based on the International Agency for Research on Cancer (IARC), obesity is associated with an increased risk of many cancers such as colorectal, kidney, esophagus, endometrium, breast, pancreas, thyroid, liver, ovary, gallbladder, and prostate cancer. The mechanisms underlying this association are multiple but especially related to elevated levels of free circulating hormones (insulin and estradiol) and their impact on hormone-dependent cancers, such as breast and prostate (96). Gender differences in obesity-related cancers, therefore, need to be considered. The estimate of the obesity-related global cancer burden, expressed as population attributable fraction (PAF), is 11.9% in men and 13.1% in women. In men, the largest PAF is observed for esophageal adenocarcinoma, while in women for endometrial cancer. Overall, obesity-related cancers affecting both genders show a higher incidence in males: concerning gastrointestinal cancers, BMI was positively associated with colorectal cancer only in men, while the incidence of esophageal adenocarcinoma and hepatocellular carcinoma (HCC) is higher in men than women with obesity (97). A role seems to be played by the decreased levels of adiponectin and the increased secretion of proinflammatory adipo-cytokines such as leptin, resistin and IL-6 in males with obesity. Indeed, they can promote initiation, progression, and metastasis of tumors, with the exception of adiponectin, which has anti-carcinogenic effects (98).

On the contrary, gallbladder cancer is more frequent in women, probably due to hormonal factors, such as high estrogen exposure. Increased risk of cancer-death per 5 kg/m^2^ BMI increase was found for hepatocellular carcinoma, intra-, and extrahepatic cholangiocarcinoma in women (99). It is of interest to rule out as a higher risk of gynecological cancer (+35% for ovarian cancer, +19% for endometrial cancer and +5% for breast cancer) has been recently reported in a large cohort of cholecystectomized women (100).

Pancreatic cancer is another tumor associated with obesity in both genders, although its incidence is slightly higher in men, where a strong link with fasting hyperinsulinemia has been reported Regarding renal cell cancer, although epidemiological studies report that males have a double greater risk of developing kidney cancer and a higher death rate than females, a meta-analysis indicates a stronger association between renal cancer and increase in BMI in in women (34%) than men (24%) (101).

In men, besides prostate cancer risk, a 5 Kg/m^2^ increase in BMI has been associated with a 15% increase in prostate cancer mortality and a 21% increased risk of its biochemical recurrence. Although some studies have shown a consistent effect of androgens on prostate cancer, the subsequent in-depth analysis showed no associations between the risk of prostate cancer and sex hormones, unlike other mechanisms such as inflammatory cells that play important roles in tumor progression via adipose-secretory cytokines or chemokines (102).

Thyroid cancer is 5-fold more common in women than men, suggesting a key role for levels of endogenous estrogens, acting as a growth factor for benign or malignant thyroid nodules. Other putative mechanisms include insulin resistance, IGF-1, adipo-cytokines, TSH, sex hormones as well as chronic subclinical inflammation (102). However, the relationship between thyroid neoplasms and sex appears complex, and a recent pooled analysis from the Asia Cohort Consortium has shown that, although the overall thyroid cancer risk was lower among underweight men and women, the papillary cancer risk may be elevated among underweight men (103).

Among obesity-related cancers in females, a detrimental effect of obesity on the risk of hormone-sensitive breast cancer in postmenopausal women has been provided by multiple studies. About 7% of all postmenopausal breast cancer can be related to overweight/obesity (102), probably due to the increased levels of estrogens due the increased aromatase enzyme activity. As a matter of fact, the increase in breast cancer risk is decreased by the reduction of serum estrogen concentrations.

However, obesity may contribute to malignant progression through other mechanisms, being adipose tissue able to produce bioactive molecules like cytokines, chemokines and vascular growth factors which play integral roles in mediating physiological processes and influencing cancer-related pathways. Inflammatory mediators crosstalk with cells by acting on surface receptors, activating inflammatory signaling pathways, and modifying the behaviour of the tumor, the immune system and the stromal cells (104). Targeting dysfunctional obese breast adipose tissue through weight reduction or pharmacological approaches might help in decreasing breast cancer risk; however, more intervention trials and well-designed observational studies in diverse populations are needed to elucidate the impact of body weight and composition and their changes on breast cancer outcomes (105).

### 4.6 Knee osteoarthritis and osteoporosis

Obesity is associated with comorbidities of the musculoskeletal system, such as osteoarthritis (OA) and osteoporosis (106). Obesity-related OA is a degenerative joint disease that can affect any joint, even though knee osteoarthritis (KOA) is the most common form. OA is characterized by progressive deterioration of the articular cartilage, subchondral bone remodeling, and synovial membrane inflammation (107). This complex condition substantially impacts patients’ quality of life, and clinical and animal studies have revealed that its incidence increases with increasing BMI (108). Notably, the KOA prevalence in patients with obesity is around 30.6% compared with 16.4% in normal weight people, but in women this difference is even more evident in women with obesity being 4-6 times higher than normal weight women (109). Even more, BMI >27 Kg/m^2^ accounts for 25% of cases of KOA in women, also with greater severity, against the 20% in men (109).

From a pathological point of view, it is believed that structural damage to the joint derives from both mechanical and metabolic factors. The excessive mechanical load on the joint and muscle weakness due to overweight contributes to the osteoarthritic joint process, including joint deformation, instability, and loss of cartilage homeostasis (108). On the other side, an important role seems to be played by obesity-induced inflammation. Indeed, adipokines may exert detrimental effects on joint tissues, as suggested by the expression of leptin receptors on the surface of chondrocytes, synoviocytes and subchondral osteoblasts and by its ability to increase degradative enzymes levels, such as matrix metalloproteinases (MMPs) (110); also the concentration of proinflammatory citokynes, such as IL-6, has been found increased in the synovial fluid and serum of OA patients and correlated with the incidence and severity of the disease (111). In addition, the presence of estrogen receptors in chondrocytes and synovia (112) together with the observation of a more steeply rise in KOA incidence in women close to the age of menopause than in men suggest a protective role of estrogens in joint health (113).

Estrogen receptors are also crucial in regulating bone turnover (112). Indeed, osteoporosis, which is characterized by low bone mineral density and bone microarchitecture alteration with a consequent increased risk of fracture, although affecting both sexes, most commonly occurs in postmenopausal women. However, besides the well-known direct negative effects of estrogen-deficiency on bone (106), the increased visceral adiposity after menopause, by inducing a pro-inflammatory phenotype with altered cytokine expression and immune cell profile, may detrimentally contribute to induce osteoblast apoptosis and osteoclastogenesis (114).

## 5 Cardiovascular and renal complications in obesity

### 5.1 Obesity and cardiovascular risk

Obesity has been recognized as an established risk factor for cardiovascular disease and heart failure for decades; several factors mediate this detrimental relationship. Visceral adiposity has emerged as a better predictor of cardiometabolic risk than BMI: visceral fat deposition, indeed, promotes metabolic and pro-inflammatory abnormalities able to enhance oxidative stress, endothelial dysfunction and insulin resistance (115). However, women’s pattern of fat distribution varies according to the different stages of life. Premenopausal women have more subcutaneous adiposity and less visceral adiposity than men (116); they tend to accumulate more fat in the gluteus–femoral area (the “ginoid” phenotype) and often remain metabolically healthy. In women with overweight and obesity gluteus–femoral fat has been found to play a protective effect on glucose and lipid-related cardiometabolic risk, and associated with a beneficial adipokine profile and fewer pro-inflammatory molecules compared with subjects with prevalent visceral fat distribution (117, 118). Gluteus–femoral fat also exerts protective vascular effects: indeed it is associated with lower aortic calcification and arterial stiffness (119), as well as with a decreased progression of aortic valve calcification in women (120). Young women also show a greater prevalence of brown adipose tissue (BAT), which affects energy metabolism and is inversely related to age and BMI (121).

Detrimental mechanisms that worsen the cardiovascular risk profile of women during the peri-menopausal transition are related to the rise in visceral, perivascular and epicardial adiposity due to the decline in estrogen levels (122–124). Particularly, perivascular and epicardial fat are strongly associated with coronary and abdominal aortic calcium independently from traditional measures of obesity playing a possible direct role in the higher risk of CVD reported after menopause (125, 126). In addition, epicardial fat may adversely affect cardiac muscle function and remodelling with a possible pathogenetic role in heart failure and arrhythmias (127, 128). This highlights the importance of specific management strategies aimed at promoting weight loss and targeting visceral fat stores.

In recent years, sarcopenic obesity, a functional and clinical condition characterized by the coexistence of loss of skeletal muscle mass and function and an excess of adipose tissue, has raised attention as a major determinant of the increased cardiovascular risk profile of individuals with obesity, as well as of several other clinical complications such as frailty, reduced bone mineral density and fractures, cancer, and an increased risk of hospitalization and all-cause mortality (129, 130). This may be ascribed to a more prominent loss of muscle and bone mass with increasing age and a greater increase of visceral fat following menopause in women compared with men of similar age (131, 132). Targeting health behaviours, such as dietary salt reduction, smoking cessation, increasing physical activity/exercise training, and reducing caloric intake, is critical for fighting sarcopenic obesity-associated morbidities and mortality (133).

### 5.2 Obesity and renal risk

Obesity and chronic kidney disease (CKD) represent major health problems worldwide. Improvements in socioeconomic conditions have contributed to the rise in obesity rates (134), while the prevalence of CKD increases with aging (135). Population-based observational studies have established a significant association between obesity and the development and progression of CKD (136, 137); this configures a true clinical challenge, being both independently associated with increased rates of cardiovascular disease and all-cause mortality. In a systematic review and meta-analysis investigating the combined effects of BMI and metabolic status on CKD risk, individuals with obesity showed a higher risk of CKD compared to metabolically healthy normal-weight individuals, even in the absence of remarkable metabolic abnormalities (138).

In a classic view, the chronic renal complication of obesity has been defined as obesity-related glomerulopathy, a glomerular disease characterized by glomerulomegaly presenting alone or with focal and segmental glomerulosclerosis (139). However, the pathophysiology of kidney impairment in obesity is complex, with the contribution of abnormal renal haemodynamics, inflammation, and metabolic derangement. It has been hypothesized that insulin resistance increases renal blood flow (140) leading to hypertensive nephrosclerosis, and demonstrated that renal plasma flow and glomerular filtration rate (GFR) are elevated in individuals with obesity, suggesting a state of renal vasodilation (141). Increased activation of the renin-angiotensin-aldosterone-system (RAAS) in obesity due to sympathetic stimulation and synthesis of adipokines by visceral fat causes afferent arteriolar dilation and efferent arteriolar vasoconstriction. These factors together with physical compression of the kidney parenchyma by visceral fat increase intra-glomerular pressures, which contribute to hyperfiltration, glomerular hypertrophy, focal glomerulosclerosis, and proteinuria. In obesity, the production of pro-inflammatory adipocytokines causes chronic low-grade systemic inflammation and oxidative stress; in detail, increased leptin may promote renal fibrosis and glomerulosclerosis, while reduced adiponectin is a determinant of albuminuria development (139). Therefore, adipose tissue inflammation in obesity is multifactorial and drives renal dysfunction through a bidirectional crosstalk. CKD reduces subcutaneous fat volume, promoting the redistribution of lipids to ectopic depots with subsequent lipotoxicity (142). Indeed, ectopic renal lipid deposition also occurs in CKD, increasing renal inflammation.

Another peculiar aspect influencing CKD onset and progression in individuals with obesity is the presence of the OSA.

The renal medulla, whose blood flow is tightly regulated to maintain the interstitial medullary osmotic gradient, is highly vulnerable to ischemic injury. Hypoxia in OSAS patients over-activates the sympathetic and RAAS systems, which are associated with long-term renal impairment (143). Longitudinal studies have revealed that nocturnal hypoxia is independently associated with faster declines in eGFR, cardiovascular and all-cause mortality (144, 145). A quite recent observation performed in a large cohort from Taiwan has shown that OSAS accelerates kidney dysfunction in subjects without hypertension and diabetes, particularly in women (146).

In exploring the relationship between gender and CKD, several factors influencing gender-related disease progression might be proposed. First, GFR-estimating equations may perform better in men than women, possibly overestimating the prevalence of the disease in women. Second, due to hormonal differences, women may be protected from kidney disease earlier in life, a protection that may wane after menopause. Third, the higher health awareness of women often plays a role, with women undergoing more frequently screening programmes in comparison to men. Also, in traditionally hierarchical societies, women may not have equal access to health care (147). In women, waist circumference mirrors visceral adipose tissue better than in men, and visceral adipose tissue accurately predicts CKD in women, thus representing a reliable sex-related cardiometabolic risk marker. However, data disaggregated by age, sex, or obesity are still scarcely available from prospective studies (148, 149), and further large-scale studies are required to solve the existing controversies, especially concerning sex-specific kidney disease prognosis and establishing research supporting precision/personalized medicine (150). Similarly, clinical trials must ensure adequate representation of both genders and acknowledge potential effects of sex or sex-specific treatments when assessing outcomes.

### 5.3 Glomerular hyperfiltration

Glomerular hyperfiltration (GH), a phenomenon seen in several clinical conditions like obesity and diabetes, is recognized as the initial stage of kidney damage. There is a growing understanding that mechanisms behind GH may vary between men and women. In T2D, when the progression of diabetic kidney disease was monitored over time, women showed a three-fold greater risk than men of developing GH over five years, while adolescent girls with T1D had a four-fold increased prevalence of GH compared to males of similar age and glycemic control (151).

In US youth/young adults, prevalence of GH increases from 6.5% to 11.8% moving from class 1 to class 3 obesity; factors associated with an increased prevalence of hyperfiltration in the bivariate analysis included elevated ALT, non‐White/non‐Hispanic races/ethnicities, and female sex (152).

A recent cross-sectional observation of 62,379 non-diabetic individuals from a Japanese health insurance database shows that BMI and GH are linearly related in women, while a U-shaped relationship is evident in men (153).

Of note, the origin of the obesity-related hyperfiltration is the increased proximal tubular sodium (and glucose) reabsorption via sodium-glucose cotransporter 1 and sodium-glucose cotransporter 2 (SGLT1 and SGLT2). The inhibition of the SGLT2 in the proximal segments of the nephrons is an ideal intervention to inhibit the tubulo-glomerular feedback and mitigate glomerular hyperfiltration in subjects with obesity, thus representing an important breakthrough in reducing the consequences of this phenomenon, as well as a more accurate evaluation of anthropometric measurements (waist-to-height ratio or, when available, DEXA or segmental impedance).

The increased expression of angiotensin II receptor (AT1R) induced by estradiol, a female hormone, might explain the different gender prevalence of GH in subjects with diabetes. Hyperglycemia activates the RAAS and elevates angiotensin II levels, leading to a higher efferent arteriolar resistance observed in T1D women (154). Interestingly, although GH has been shown to worsen the progression of end-stage kidney disease (ESKD) by speeding damage at the level of the filtration barrier, it should be noted that advanced renal complications tend to appear more than a decade later in women (155). Thus, women are more prone to developing ESKD, but the overall progression of the disease is slowed and often aligns with the woman’s transition into the postmenopausal phase (155). These changes have been partly linked to the differences in gene expression and sex hormones signaling within the kidney that contribute to regulating GFR (156).

In a recent animal study, it was observed that both gender mice developed hyperfiltration and albuminuria, but these changes were more pronounced in ovariectomized females, which had also higher IL-1β and TNF-α compared with not-ovariectomized counterpart and male mice (157). This suggests that the absence of estrogen hormones, particularly in the context of obesity, may intensify the detrimental effects of excessive lipid accumulation and inflammation (158). Additionally, estrogens influence the renal blood flow through the expression of endothelial nitric oxide synthase and the sodium reabsorption in proximal tubular cells, providing an additional protective function (151).

### 5.4 Biomarkers/risk predictors of CKD in subjects with obesity

In the last decade, attention has been paid to searching for easily measurable biomarkers able to predict CKD onset and progression during metabolic diseases; indications from such scientific literature seem, so far, partially inconclusive, at least for obesity.

Recently, population-based data from 100,269 Austrian individuals suggest that TyG index, mean arterial pressure, and uric acid, but not total cholesterol, mediate the association of BMI with end-stage kidney failure in middle-aged adults (159). Indeed, uric acid is a promising biomarker for future weight gain and cardiometabolic risk in young adults that may respond to weight gain prevention (160).

Another suggested biomarker is remnant cholesterol, independently associated with an increased risk of prevalent CKD in a general middle-aged and elderly Chinese population, especially in women, subjects with overweight/obesity, and without CVD events (161).

A report putatively able to impact daily dietary habits is that high-salt intake was particularly associated with a higher risk of composite renal outcome in women, in patients <60 years of age, in those with uncontrolled hypertension, and in those with obesity (162).

Lastly, markers of systemic subclinical inflammation, like CRP and IL-6, that might accelerate CKD progression correlate with measures of adiposity, and this association is stronger in women than in men for all measures of adiposity as well as the entity of visceral adiposity (163).

## 6 Psychological characteristics associated with obesity

The main psychological problems observed in individuals with obesity seem to be largely the consequence of an adverse social environment, which exerts strong discrimination against those with a body size above average. Psychological problems and associated cognitive processes can contribute to the maintenance and worsening of obesity by hindering its treatment and favoring the adoption of an unhealthy lifestyle.

This section of the review describes the main psychological constructs associated with more frequency in the female gender with clinical relevance to patients’ quality of life and treatment.

### 6.1 Weight bias, weight stigma, and weight stigma internalization

Weight bias is a term used to define negative attitudes towards people perceived to have higher body weight. These irrational attitudes are manifested by stereotypes and/or prejudices towards these people who are considered lazy, unintelligent, unattractive, and lacking willpower and self-discipline. At the root of these attitudes is a moral judgment that blames the people with higher body weight for being responsible for their condition (164).

Negative stereotypes towards people with higher weight are rarely challenged by Western society, which favors the development of the weight stigma, the societal devaluation, or discrediting due to weight (164).

The weight stigma produces actions against people with higher weights or larger body sizes that cause exclusion and marginalization and lead to inequalities. The weight stigma is promoted at multiple levels: (a) structural (media, laws & policies); (b) interpersonal (teasing/bullying, discrimination); and (c) intrapersonal (anticipation, internalization) (165).

Both men and women are vulnerable to weight discrimination. However, women, in particular those who are middle-aged or with lower levels of education, experience a higher rate of weight stigmatization than men, even at lower levels of excess weight (166).

Internalized weight stigma occurs when a person has negative beliefs about themselves (e.g., I feel lazy, I don’t have willpower, I hate myself for not having self-control, I feel like a failure, I feel inferior to those who can control their weight; I am ugly and disgusting). Internalized weight stigma can be assessed with the Weight Bias Internalization Scale (WBIS- 10 or 11 items, 1-7, averaged) (167), and/or the Weight Self-Stigma Questionnaire (WSSQ - 12 items, 1-5, summed) (168).

Women report a higher level of weight bias internalization than men, and younger participants have more internalized weight bias than older participants (169). In women, a correlation exists between a higher BMI and a higher level of internalized stigma (170).

A recent study, which evaluated 13,996 adults who sought treatment for obesity in six countries (171), found that, by controlling for participant characteristics and weight stigma experiences, women reported higher weight stigma internalization than men. No significant differences in WBIS-M scores were found based on race/ethnicity, education, or sexual orientation. WBIS-M scores were associated with more significant weight gain over the past year. Participants with higher WBIS-M scores also reported poorer mental and physical quality of life, lower self-efficacy in eating control and physical activity, more frequent use of food as a coping strategy, greater avoidance of gym attendance, worse body image, and higher perceived stress. Recent studies in which the majority of participants were women have also found an association between internalised weight stigma and metabolic syndrome (172), low self-efficacy (173), and eating disorder psychopathology (174).

### 6.2 Overvaluation of weight and shape

While most people evaluate themselves based on their perceived performance in a variety of domains of their lives (e.g., school or work performance, quality of interpersonal relationships, skills in a particular sport or hobby, etc.), some judge themselves predominantly, sometimes exclusively, in terms of shape, weight, shape, and their control. This form of self-evaluation, termed overvaluation of weight and shape,” is considered the core psychopathological feature of eating disorders, such as anorexia nervosa, bulimia nervosa and other similar states, and in about 50% of individuals with binge-eating disorder (175), which affect a significant larger proportion of females than males (176), because most of the other features observed in these disorders derive directly or indirectly from it (177). However, the overvaluation of weight and shape it has been also observed in about 20% of those seeking treatment for obesity without eating disorders (178).

The overvaluation of weight and shape should be distinguished from body dissatisfaction, a term used when a person does not like their physical appearance. Body dissatisfaction is widespread among people and does not necessarily represent a clinical problem because it is less associated with self-evaluation and, generally, does not impair, as does the overvaluation of weight and shape, the quality of life of people (179).

The overvaluation of weight and shape indicates the severity of binge-eating disorder because it is associated with a higher frequency of binge-eating episodes and more severe psychological distress and psychosocial damage (175). In addition, in people with obesity, it is more frequently reported by women than men (70.6% versus 29.4%), and it is associated with higher weight loss expectations, more severe eating disorder psychopathology, higher general psychopathology, and worse mental quality of life (178).

### 6.3 Low self-esteem

Self-esteem refers to how people perceive and value themselves. In a more elaborate form, it is “the extent to which a person believes himself to be capable, significant, successful and worthy.” (180) Several data indicate that low self-esteem has harmful, sometimes devastating effects on the person and their life. Indeed, low self-esteem is associated with negative thoughts about oneself (self-criticism), negative emotions (depression and anxiety), physical symptoms (fatigue, low energy, or tension), problematic behaviors (avoiding challenges or taking excessive precautions) that impair school or work performance, interpersonal relationships, leisure, and self-care (181). It has been also found that girls with overweight tend to report lower levels of self-esteem compared to boys who are overweight (182). Depending on how it is assessed, a distinction can be made between global self-esteem and specific self-esteem based on competence in externally (and internally) valued domains and as a metric of social acceptance (or likely rejection) (180). Both global and specific self-esteem should be considered when evaluating this construct’s relationship with obesity.

Global self-esteem, which concerns an overall judgment of one’s worth, is a construct attributed to Rosenberg, and the questionnaire he developed, the “Rosenberg Self-Esteem Scale,” is considered the gold standard in self-esteem research (183). Unsurprisingly, this questionnaire has been the most widely used in obesity research. A meta-analysis examining global self-esteem across all age groups found an effect size of -0.36, indicating a robust but small to moderate relationship (184). Scores in people with obesity are significantly influenced by age and gender. Indeed, the correlation between weight and self-esteem increased from -0.12 to -0.22 and -0.28, respectively, in children, adolescents, and young college-age adults, and the relationship is stronger in females (-0.23) than in males (-0.09). A more recent systematic review confirmed the relationship between global self-esteem and obesity in 17 of the 21 included studies (185). The four exceptions concerned studies carried out in samples from Asia or minority groups in the USA.

A review of studies assessing a specific form of self-esteem dependent on competence in various areas found that young people with obesity score lower in physical appearance and athletic/physical competence. In contrast, in comparison with peers without obesity, few differences were found in academic competence and behavioral conduct (186). However, there were no clear differences in effects between children and adolescents, and evidence on gender and ethnicity was lacking.

A study performed in the USA in a community sample of children aged 6-7 years assessing the specific form of self-esteem dependent on the degree of social acceptance or rejection found that those with obesity were more likely to be neglected than those of normal weight, having few positive or negative “nominations” from peers (187). A meta-analysis of 16 studies found a significant relationship between having an obesity condition and being victimized (OR = 1.51) in children aged 11 years and older (187). A study revealed that children with obesity were more likely to be victimized by their peers but also more likely to bully others (188).

Most studies evaluating the effects of weight-loss interventions on self-esteem in children and adolescents have reported improvements in global self-esteem (189) and the most affected skills, namely physical appearance, athletic competence, and social acceptance (190). However, it seems that individual (self-efficacy, motivation), interpersonal (family and friends), and institutional (place of residence, school, and workplace) resources are even more important than weight loss in influencing self-esteem (180). Many people with obesity, both adults and children, have high self-esteem, do not suffer from severe depression, have a well-paid job, and have good social relationships. This implies individual resistance or resilience and the key importance of implementing a resource-based rather than deficit-based approach to health improvement (180). However, it is not easy to identify and develop resources external to the individual in an environment characterized by anti-fat attitudes.

### 6.4 Cognitive processes

The “complex behaviors” in weight loss and maintenance through lifestyle modification are influenced by conscious cognitive processes. It is, therefore, plausible that these may play an influential role in an individual’s success or failure to maintain lost weight. Cognitive factors have been largely neglected in traditional obesity treatments, even if basic scientific research demonstrates the critical role of cognitive processes in maintaining unhealthy eating habits and making healthier eating difficult (191). This is also supported by the results of numerous clinical trials that have, respectively, shown associations between specific cognitive factors and treatment discontinuation, as well as the amount of weight lost and long-term weight maintenance lost (192).

For example, studies in Italy indicate that people who start weight loss treatment have an average weight loss expectation of 32%, and women have lower maximum acceptable BMI, lower dream BMI, and higher expected 1-year BMI loss than men (193). People seeking obesity treatment often have “primary weight loss goals” to improve interpersonal relationships, self-confidence, finding a partner or a new job, and not to improve health.

Available data indicate that these goals are unrealistic because there are no obesity treatments, including bariatric surgery, that can determine an average weight loss of more than 30% in the long term. Furthermore, many non-health primary goals are often not achievable even with a large amount of weight loss.

Unrealistic weight and primary goals seem to be associated with treatment discontinuation (194, 195) or not maintaining lost weight because people consider the result unsatisfactory (192). In addition, people who have unrealistic weight goals often intermittently adopt a dysfunctional dietary restriction that contributes to triggering and maintaining binge eating episodes (192).

### 6.5 Events, associated mood changes, and eating

Overeating and binge eating are often triggered and maintained by life events and associated mood changes (196). The main mechanisms that explain the association between life events and mood changes are as follows: (a) food can be used to distract from adverse events and worrying problems; (b) food can help to cope with emotional states; (c) food can be used to create positive emotions (177). The episode of overeating, after an initial attenuation of the negative emotional state, in certain people often produces feelings of guilt, anxiety, and low mood, which, in turn, can trigger a new episode of uncontrolled eating.

A subgroup of people, particularly those in whom obesity coexists with binge-eating disorder, a disorder that is more common in women than men (176), also have a coexisting problem of “mood intolerance.” This expression defines individuals who are exceptionally sensitive to emotions, with frequent negative emotions (e.g., anger, anxiety), and have difficulty tolerating and managing them. Those who suffer from this problem often adopt dysfunctional mood-modulating behaviors, such as taking psychoactive substances (alcohol or tranquilizers) or bingeing, which reduce awareness of the emotional state (and associated thoughts) entailing, however, a personal cost (emotional, interpersonal, and physical) (177).

### 6.6 Personality

Many of the early personality studies in obesity, which used the Karolinska Scales of Personality or the Minnesota Multiphasic Inventory, produced inconsistent results, but this is not surprising, as both of these tools were designed and validated to assess pathological personality traits rather than interindividual variation in normal personality traits (197).

In recent years, the Temperament and Character Inventory (TCI) (198), designed to provide a comprehensive assessment of non-pathological personality, has been widely used to study obesity and has highlighted that certain personality traits appear to play a role in influencing obesity management (199). In particular, low scores in “novelty seeking” (a temperament trait that reflects a tendency to be motivated by a desire to avoid aversive experiences) and “self-direction” (a character trait that measures self-concept, self-acceptance, and the ability to direct one’s life according to personal goals and values) at baseline appear to predict better weight loss outcomes at ≤ 6 months. On the contrary, higher “persistence” scores (a temperament trait that describes resistance to maintaining frustrating behavior) seem to predict the maintenance of weight loss (199).

A study also found significantly higher scores on questionnaires assessing binge eating and night eating in women with obesity consecutively seeking treatment at eight Italian medical centers, obesity in comparison with age-matched women in normal weight range without eating disorders (200). High binge eating scores were associated with high novelty seeking and harm avoidance and low self-directedness, the last two personality features being also associated with high night eating scores. These data confirm that personality traits differ between patients seeking treatment for obesity and controls, and the presence of disordered eating is associated with specific personality characteristics.

## 7 Treatment of obesity

### 7.1 Dietary and lifestyle interventions

Dietary and lifestyle weight-loss interventions are typically based on energy intake reduction and increased exercise, with or without changes in macronutrient balance and diet quality. However, maintaining long-term adherence to weight-loss interventions is a major issue, and rebound weight gain is commonly observed following the initial success. The effects of strategies combining diet with exercise have been estimated in a recent meta-analysis (201), suggesting a moderate-to-poor long-term success. A recent growing interest in intermittent fasting practices, like time-restricted feeding (TRF) that confines daily food intake to 6 to 10 hours with no calorie restriction, is observed, although the evidence-base for TRF as an intervention for obesity is, so far, relatively sparse. Moreover, before prescribing TRF, clinicians should accurately evaluate the risk of precipitating the development of disordered eating with this eating pattern, especially in the young (202).

Females appear more health-orientated, motivated to lose weight, and more frequently participate in weight loss studies (203). Commercial weight loss programs are considered “female-focused,” reporting low participation of males (204). Reasons for such disparity could be insufficient motivation to lose weight in males or that females experience more social pressure.

Any nutrition intervention should be tailored to consider personal values, preferences, and social determinants of eating habits. According to the most recent European Association for the Study of Obesity (EASO) Position Statement on medical nutrition therapy for the management of overweight and obesity, several nutrition interventions, including the Mediterranean diet, vegetarian diets, Nordic and low-carbohydrate diets, among others, have been proven to affect positively metabolic parameters with or without weight loss (205). Recent evidence also supports partial meal replacements to improve weight loss and body composition outcomes compared to traditional lifestyle interventions (206).

A metanalysis of 58 weight loss intervention studies based on different diet and exercise prescriptions showed that men lose more absolute and percent body weight (possibly due to their greater baseline weight) in response to the same lifestyle modification (207). Moreover, weight loss affects metabolic outcomes differently in males and females, with males showing a better response in terms of intra-abdominal fat loss and improvements in the metabolic risk factor profile (208). However, very few studies have explored gender differences with one specific dietary regimen, and most of those that did lacked supporting data.

### 7.2 Pharmacologic treatments

Besides dietary and behavioural changes, other non-surgical interventions include weight loss pharmacotherapies. Metformin has not been officially approved as a weight-loss promoting drug because its effect on different populations remains inconsistent, although metformin is often used to improve insulin resistance also in non-diabetic subjects with obesity. and hyperandrogenism in women with PCOS (209). Orlistat exerts a peripheral effect consisting in the inhibition of gastric and pancreatic lipases with consequent dietary fat absorption decrease and moderate weight loss (210). A combination of the dopamine and norepinephrine reuptake inhibitor bupropion and the opioid antagonist naltrexone, fosters satiety while decreasing food craving (211, 212). Naltrexone, an opioid antagonist with indication for the treatment of opioid and alcohol dependence, inhibits the appetite-enhancing effects of beta-endorphin caused by cannabinoid-1 receptor activation decreasing food cravings in subjects with obesity and binge-eating (213). Bupropion stimulates POMC neurons in the hypothalamus to secrete α-melanocyte stimulating hormone that has anorexic properties, and when combined with naltrexone, it has been shown to alleviate addictive over-eating and to display a synergistic effect on appetite suppression, thus inducing a consistent weight loss, even in the face of high dropout rates (about 45%) (214). However, a recent study did not demonstrate effectiveness for reducing binge eating, although it showed effectiveness for weight reduction in these subjects (215). Adverse effects of such a combination include constipation and dry mouth, headache, insomnia, and anxiety.

Obesity increases the incidence of depression, and the prevalence of depression is approximately double for women with obesity compared to their male counterparts (216). Therefore, the potential anti-depressant effects of this medication might offer dual benefits for this population. In addition, estrogens seem to enhance bupropion antidepressant activity and desensitize μ-opioid receptors in hypothalamic POMC neurons (217). It should also be considered that sex steroids influence the pharmacokinetics of both naltrexone and bupropion, being estrogens a powerful promoter of bupropion hydroxylation and androgens potent inhibitors of naltrexone liver hydroxylation.

GLP-1 receptor agonists (GLP-1RAs) are a class of antidiabetic drugs that, in addition to hypoglycemic effects, have demonstrated the ability to reduce body weight as well as important protective effects at the CV and renal level in individuals with T2D and obesity (218).

In 2014, the Food and Drug Administration (FDA) approved the GLP-1RA liraglutide for chronic weight management for its capacity to decrease appetite and enhance satiety, presumably through effects on the central nervous system and inhibition of gastric emptying. In an early study, patients with overweight or obesity received daily liraglutide (up to 3.0 mg s.c.) or placebo or orlistat for twenty weeks (219). More individuals (76%) lost more than 5% weight with liraglutide 3.0 mg than with placebo (30%) or orlistat (44%). To compare weight maintenance in overweight patients without diabetes after an average of 6% weight loss with energy-restricted dieting, subjects were randomized either to liraglutide (3.0 mg daily) or to placebo (220). During this maintenance period, the weight loss with liraglutide was 6.2%, and with placebo 0.2%. Nausea, vomiting, and diarrhea may occur as side effects of liraglutide. The SCALE trial followed 3731 patients with obesity receiving liraglutide; over 56 weeks, 63.2% and 33.1% of all participants significantly lost at least 5% and 10% of their body weight, respectively. This trial comprised 78% of women with an average age of 45 (221). A drawback with this drug is that it requires to be administered by daily subcutaneous injections. Liraglutide may improve human fertility, particularly for women with PCOS (222). However, information on putative gender-related differences in efficacy and safety of liraglutide is scanty, and large, well-conducted real-life studies would be helpful.

### 7.3 Novel anti-obesity drugs

#### 7.3.1 Semaglutide

The main objectives of weight management are to achieve a clinically significant and sustained weight loss, minimizing weight regain to prevent the progression of T2D and other obesity-related complications (223). Weight loss of 5% or more of initial body weight improves the obesity-related complications, while a higher weight reduction produces greater overall health benefits. New drugs for treating obesity, combined with lifestyle changes, have demonstrated their important effectiveness in facilitating weight control (224). Among the GLP1-RAs, semaglutide is a potent long-acting analog that requires once-weekly administration and has been shown to reduce energy intake and hunger and increase feelings of satiety and fullness (225). In 2021, after the publication of the results of the studies STEP (Semaglutide Treatment Effect in People with Obesity) 1–4, the FDA approved semaglutide 2.4 mg for weight control in people with overweight (and comorbidity) or obesity. Specifically, it was demonstrated that semaglutide 2.4 mg resulted in significant and sustained weight loss at 68 weeks, with an approximate reduction from baseline weight of 14-16% in participants without T2D and 9.6% in those with T2D, along with improvements in cardiometabolic risk factors and with a safety profile (218). STEP 5 (Semaglutide Treatment Effect in People with Obesity) confirmed the reduction of 15% of body weight in people with obesity after two-years of treatment (226). The STEP studies also confirmed that people with T2D have more difficulty losing weight than individuals without T2D, also showing a gender difference, with a better response of women to treatment compared to men (227). Furthermore, the results published from the study SELECT (Semaglutide Effects on Heart Disease and Stroke in Patients with Overweight or Obesity), in which weekly treatment with semaglutide 2.4 mg in not diabetic patients with overweight/obesity and established CVD disease resulted in the reduction of the 20% risk of heart attack or stroke. Then, the multiple potential of semaglutide, initially developed for diabetes and weight management, has now expanded to include the reduction of CV events (228). Globally, these observations encourage individualization of obesity pharmacotherapy by exploring differences based on gender and comorbidities, such as T2D and CVD risks, to optimize the potential benefits of semaglutide in these different medical contexts (227) as well as many other drugs able to obtain a more marked reduction in body weight.

#### 7.3.2 Tirzepatide

Tirzepatide is a novel and first-in-class glucose-dependent insulinotropic polypeptide/glucagon-like peptide 1 receptor agonist (GIP/GLP-1RA), approved in 2022 by FDA and in 2023 by the European Medicines Agency (EMA) as an adjunct to diet and exercise for the treatment of T2D, and in 2023 for chronic weight management by FDA (229, 230).

The effects of tirzepatide on glycaemic control are mediated not only by improvements in glucose-dependent insulin secretion and reduced fasting and meal-stimulated glucagon levels (231) but also by significant body weight loss. Indeed, activation of both GLP-1 and GIP receptors in the CNS also appears highly effective in reducing appetite and food intake (232–234) and, in preclinical studies, high-caloric and fat diet preference (235). Tirzepatide has been suggested to promote thermogenesis in BAT in murine models (236). However, a clinical trial to assess energy expenditure utilizing respiratory chambers is ongoing (237).

In the phase 3 clinical program (SURPASS), designed to assess the efficacy and safety of once-weekly subcutaneously injected tirzepatide (5, 10, and 15 mg), as monotherapy or combination therapy, tirzepatide demonstrated superior glycemic and body weight control compared with placebo and active comparators across a broad spectrum of patients with T2D (238–243), irrespective of gender (244, 245).

Tirzepatide also induced other cardiovascular benefits by improving the lipid profile and reducing blood pressure, visceral adiposity, and intra-hepatic triglycerides (238–243, 246).

Although the potential for tirzepatide to improve CV outcomes is currently being assessed in a CV outcomes trial (SURPASS CVOT), preliminary evidence for CV safety has been provided by the SURPASS-4, a trial recruiting subjects with known CVD or at high CV risk (241), and by a metanalysis covering the whole clinical trial program (247). Similarly, kidney-protective effects were suggested by a prespecified and *post-hoc* analysis of the SURPASS-4 trial (248). Subgroup analysis for the primary outcomes by sex did not differ from overall results (247, 248). However, subjects achieving higher categorical body weight (>15%) were more likely to be women (249).

The efficacy and safety of tirzepatide have been evaluated in weight reduction and maintenance in adults with obesity in the SURMOUNT trials (229), whose initial data support greater efficacy for clinically meaningful weight reduction beyond that achieved with agents currently approved for obesity (250) (251, 252). Indeed, in the SURMOUNT-1 trial, the mean weight reduction was -15.0% with 5 mg, -19.5% with 10 mg, and -20.9% with a 15 mg weekly dose compared to -3.1% with a placebo. The percentages of individuals with a substantial weight reduction of 5% or more were 85%, 89%, and 91% with tirzepatide 5 mg, 10 mg, and 15 mg, respectively (250). Instead, when considering a reduction in body weight of 25% or more, 15%, 32% and 36% of participants in the 5 mg, 10 mg and 15 mg tirzepatide groups, respectively, met this target, as compared with 1.5% of participants in the placebo group (250).

The tolerability profile of tirzepatide was similar to what has been reported for selective GLP-1RAs, with most of the adverse events being gastrointestinal, mild-moderate in severity, and occurring during dose-escalation.

#### 7.3.3 Lifestyle modification and intensive behavior therapy associated with the new medications to treat obesity

New medications to treat obesity should always be associated with lifestyle modification. This recommendation is based on several considerations. First, STEP and SURMONT trials associated semaglutide and tirzepatide with a lifestyle modification intervention. The approach included regular lifestyle counseling sessions delivered by a dietitian or a qualified health care professional to help the participants adhere to healthful, balanced meals with a moderate caloric deficit (e.g., 500 calories per day) and physical activity (e.g., at least 150 minutes of per week) (250, 253), often with the help of basic behavior procedures such as the daily recording the diet and activity within a diary or using a smartphone application (253). Second, a healthy eating pattern and physical fitness are associated with reduced mortality and cardiovascular diseases, even without significant weight loss (254, 255). Third, regular physical activity helps reduce the loss of fat-free mass during weight loss (254, 256). Finally, regular counseling with a specialist in lifestyle modification and obesity can help the patients maintain their motivation and develop specific skills to address the inevitable obstacles during the long weight loss and maintenance process (257).

An unanswered question, however, is whether intensive behavioral therapy (BT) with an initial low-calorie and meal-replacement diet or specialist cognitive behavior therapy (CBT) for obesity are still necessary to help the patients achieve the long-term weight loss obtained by semaglutide and tirzepatide (257). For example, the STEP 3 randomized clinical trial associated semaglutide 2.4 mg combined with intensive behavioral therapy (i.e., 30 counseling visits) (258). The trial also includes an initial 8-week low-calorie diet 1000-1200 kcal/d provided as meal replacements, followed by a hypocaloric diet (1200-1800 kcal/d) of conventional food for the remainder of the 68 weeks. However, despite the intensive treatment, the STEP 3 trial did not achieve better weight loss outcomes than those of the STEP 1 trial in which semaglutide 2.4 mg was combined with a less-intensive lifestyle intervention program (i.e., 18 behavioral counseling visits every 4 weeks in 68 weeks) and no initial low-calorie, meal-replacement diet (253).

The above data have important clinical implications and open new scenarios for the management of obesity. First, prescribing diets characterized by severe caloric restriction and meal replacements no longer appears necessary to enhance adherence to caloric restriction and weight loss with the new medications for obesity. Second, less-intensive lifestyle and costly interventions, which can be easily disseminated in real-world treatment of obesity, appear to obtain similar results of intensive BT when both treatments are associated with semaglutide. However, further studies should confirm these findings. Third, assessing the optimal intensity and duration of lifestyle modification counseling associated with the new drugs is also needed. Finally, it will be essential to determine if specific cognitive behavior strategies and procedures associated with the medications for obesity might help the patients adopt a better healthy lifestyle and psychological wellness and reduce the attrition and/or limit the weight regain when the drug is suspended (259).

### 7.4 Bariatric surgery

Bariatric surgery, born in the 50s, has had a great boost with the advent of laparoscopy that has greatly reduced the risks that are currently superimposable to those of cholecystectomy.

For individuals with a BMI > 40 or BMI > 35 with comorbidities who are unable to lose weight, by lifestyle modifications or pharmacotherapy, standard bariatric surgery allows important and durable weight loss mainly in malabsorption procedures like Roux-en-Y gastric bypass (RYGB) and bilio-pancreatic diversion (DBP), but also in restrictive surgery as SG and adjustable gastric banding (70).

Over the years, many endoscopic bariatric modalities, including intragastric balloons, endoscopic sleeve techniques and other novel therapies have emerged as minimally invasive, cost-effective, and reversible treatments in mild to moderate obesity with no or borderline statistically significant differences in postoperative complications compared to standard bariatric interventions (260).

BS is more commonly undergone by women than men (78% vs 22%) (261). The reasons behind this inequality lie in greater health awareness and perceptions of obesity in women who more frequently pursue weight loss programs increasing the probability that women will be direct by clinicians toward BS (261).

Analyzing weight loss after BS from a gender perspective, data in the literature reported that the differences in weight loss between men and women were minimal and not significant (217). With regard to secondary outcomes, poorer psychological outcomes have been reported in women, including a worse body image, depression and lower satisfaction with the surgery itself with greater requests for post-operative hospital readmissions (262). On the other hand, women usually show better outcomes of the other obesity complications probably because they tend to be treated earlier than males (262). However, recent studies suggest that these differences in post-operative outcomes are decreasing probably for the improvement of BS procedures (263). Since the underlying mechanisms through which sex influences postoperative complications have not yet been elucidated, further studies are needed to explore gender-specific differences in bariatric surgery outcomes which may underlie differences in obesity-related comorbidities. In fact, the limited available guidelines developed by bariatric surgeons do not take in consideration the gender in the decision-making process for patient selection (264). Nevertheless, the clinicians should pay more attention to preoperative counseling of women and to the psychological effects after surgery to promptly help them to manage expectations after BS and their relationship with body image (263).

## 8 Summary and conclusions

Obesity is a heterogeneous condition with complex interactions among sex/gender, sociocultural, environmental, and biological factors. Obesity is more prevalent in women than in men in most developed countries, and several clinical and psychological obesity complications show sex-specific patterns. Females differ in fat distribution, with males tending to store more visceral fat, which is highly correlated to increased cardiovascular risk. Although women are more likely to be diagnosed with obesity and appear more motivated to lose weight, as confirmed by their greater representation in clinical trials, males show better outcomes in terms of body weight and intra-abdominal fat loss and improvements in the metabolic risk profile.

With regard to novel anti-obesity drugs, RCTs also suggest a gender difference, with a better response of women to treatment compared to men (227, 249).

Globally, these observations encourage further studies exploring gender differences in obesity and individualization of obesity pharmacotherapy to optimize the potential benefits of novel anti-obesity drugs in different medical contexts.

## Author contributions

VG: Conceptualization, Writing – original draft, Writing – review & editing, Funding acquisition. RG: Conceptualization, Writing – original draft, Writing – review & editing. FL: Conceptualization, Writing – original draft, Writing – review & editing. AS: Conceptualization, Writing – original draft, Writing – review & editing, Funding acquisition.
